# Current Progression: Application of High-Throughput Sequencing Technique in Space Microbiology

**DOI:** 10.1155/2020/4094191

**Published:** 2020-06-20

**Authors:** Yanwu Chen, Bin Wu, Cheng Zhang, Zhiqi Fan, Ying Chen, Bingmu Xin, Qiong Xie

**Affiliations:** ^1^Space Science and Technology Institute (Shenzhen), Shenzhen, China; ^2^China Astronaut Research and Training Center, Beijing, China; ^3^College of Physics and Optoelectronics Engineering Shenzhen University, Shenzhen, China

## Abstract

During a spaceflight, astronauts need to live in a spacecraft on orbit for a long time, and the relationship between humans and microorganisms in the closed environment of space is not the same as on the ground. The dynamic study of microorganisms in confined space shows that with the extension of the isolation time, harmful bacteria gradually accumulate. Monitoring and controlling microbial pollution in a confined environment system are very important for crew health and the sustainable operation of a space life support system. Culture-based assays have been used traditionally to assess the microbial loads in a spacecraft, and uncultured-based techniques are already under way according to the NASA global exploration roadmap. High-throughput sequencing technology has been used generally to study the communities of the environment and human on the ground and shows its broad prospects applied onboard. We here review the recent application of high-throughput sequencing on space microbiology and analyze its feasibility and potential as an on-orbit detection technology.

## 1. Introduction

As on earth, microbes are ubiquitous on manned spacecrafts. The experiences of a manned spaceflight in the United States and Russia prove that with the prolongation of flight time, microorganisms accumulate in the cockpit more and more seriously. On the one hand, microorganisms affect human body health as pathogens, causing infections [1] and allergies [2, 3], and their metabolites are harmful to crews; on the other hand, for the spacecraft system, some biodegradable microbes degrade the spacecraft materials and corrode instruments and effect craft hardware equipment stability or even cause system failure [4, 5]. Microbes in a spacecraft can cause serious biosafety problems.

The culture-based method is mainly used on orbit. Monitoring the total number of bacteria, pathogenic bacteria, or conditioned pathogenic bacteria in the environment through standard laboratory culture conditions and comparing it with the limit standards provide reference for biosafety assessment and on-board microbial control procedures. The cultural method has the advantages of simple and practical. At the same time, it is not restricted by microgravity environment and has lower needs for large-scale instruments and equipment. It is able to preliminarily judge strains by observing colony morphology, which to some extent meets the requirements of microbial evaluation in space missions.

However, in the space environment, the traditional cultural method has certain limitations: (1) Only a small number of microorganisms can be cultured under standard laboratory conditions, and culture-based analysis limits the comprehensive understanding of microorganisms on the space station. (2) Analysis time is long, and on-orbit culture analysis needs 2-7 days. Return to the ground analysis cannot reflect the spacecraft microbial load level in real time. (3) Due to the preference of microorganisms on the medium, experimental operation error, and other reasons, the cultural method may cause the deviation of colony count statistics. (4) Microorganisms amplificated during the culture process might be a potential biohazard.

NASA and space administration of other space powers have been looking for the uncultured technology for on-orbit microbial detection, such as the fluorescence analysis technology based on ATP levels, handheld microbial detection equipment “LOCAD-PTS” [6], gold nanoparticles [7], and miniature microfluidic PCR [8]. NASA roadmap plan mentioned to realize uncultured detection technology on orbit in 2020 (http://www.nasa.gov/pdf/500436main_TA06-HHLSHS-DRAFT-Nov2010-A.pdf). Molecular biology detection technology is considered to be the future direction of microbial detection on orbit because of its advantages of high speed and accuracy.

High-throughput sequencing can identify and quantify culturable and unculturable microorganisms, providing a more comprehensive approach to molecular evaluation. Through targeted amplicon sequencing, a specific gene (such as 16s rRNA or ITS) is detected to obtain the species and abundance of a certain group of microorganisms such as bacteria and fungi in the sample. Although sequencing technology has been shown to work on the space station, it has not been widely used for microbial monitoring on the international space station due to the special environment of the space station and the limitations of the station's payload capacity.

On-orbit sequencing technology has great potential. Real-time on-orbit detection and analysis of microorganisms can provide a comprehensive understanding of the microbial composition and changes in pathogenic microorganisms on the space station, which is conducive to the prevention of infectious diseases. The on-orbit analysis can give the infection status of bacteria or virus for the on-orbit personnel with infectious diseases and provide the basis for timely treatment. Through on-orbit metagenomic sequencing, we can also understand the situation of mutated strains in the space station in real time and prevent the growth and reproduction of adverse mutated microorganisms in time; on-orbit sequencing is of great significance to human exploration of deep space life and discovery of extraterrestrial microorganisms.

Although there is no independent on-orbit sequencing analysis facility for technical reasons currently, once a simple, compact, reliable, and microgravity suitable sequencer and sample processor are developed, they can be used for rapid, real-time microbial detection and functional analysis over long periods of time on the space station. At present, high-throughput sequencing technology has been used in ground detection of on-orbit samples and ground-based simulation experiments. It is believed that with the rapid development of science and technology, the miniaturization of sequencers and the birth of microsample processing devices will soon lead to independent on-orbit sequencing devices.

Based on the recent application of high-throughput sequencing technology in the field of microbiology, this paper analyzes the application of this technology and its feasibility and potential as an on-orbit detection technology.

## 2. Main Text

### 2.1. Space Microbiology

In a long-term manned spaceflight, the cabin of the manned space station or deep space exploration spacecraft has suitable temperature, humidity, and atmospheric environment for the growth of microorganisms. Metabolites, household wastes, food residues generated by personnel, and various materials in the cabin create a suitable external environment for the survival and reproduction of microorganisms [9]. In the spaceship's long-term closed environment, excessive accumulation, the affection of radiation, and microgravity which lead to the change of pathogens and destruction can bring adverse effect to shuttle facilities and living environment, including corrosion in the circuit board, blocking ventilation systems and water supply systems, and degradation of various materials resulting in system failure, etc. These threaten the normal operation of the space station [10].

The space station is a closed environment. Similar to the ground, the microbial community in the station is composed of culturable and unculturable microorganisms, which influence human health seriously. Caused by narrow cabin space, the microbial aerosol state changes (settling velocity decreases) because of weightlessness, and the organisms are easier to spread during the individual migrations. Also because of the weightlessness, radiation, and airtight environment, these factors may weaken the immunity of the astronauts and lead to the astronaut body dysbacteriosis, then increase the risk of individual infection. These infections may occur in the respiratory tract, digestive tract, urinary tract, skin, and other organs and affect astronaut health and subjective experience. At the same time, the migration of bacteria can also affect other individuals in the group or even the whole group. In NASA's space missions, infectious diseases caused by human-derived pathogens affecting the eye, skin, intestine, urinary tract, and other body regions occurred many times [11]. In addition, in order to minimize the harm of microorganisms to spacecraft equipment, it is necessary to take a variety of disinfection and cleaning measures in the spacecraft cockpit before launch and on orbit. But it is impossible to completely eliminate microorganisms, as the human body is the main source of microorganisms in the cockpit environment.

The early Russian Salyut space station and “Mir” space station had serious microbial pollution. The orbital time of the Salyut space station was relatively short, but due to the limited understanding of microbial pollution in the early stage of human space engineering, microbial pollution was generated [12]. The “Mir” space station was a typical long-duration flight. During its 15-year operation, microbial contamination occurred [4, 13]. 234 microbial species consisting of 108 bacteria species and 126 fungi species were successively isolated from “Mir,” including species with pathogenic and corrosive functions [4]. The international space station, currently in operation, is more stringent than ever on environmental microbial control and prevention. Investigations have found that the microbial community is similar to “Mir,” containing a variety of opportunistic pathogens and pathogenic bacteria, as well as 39 mold species in air samples, including a variety of pathogenic fungi and biocorrosive molds. The top three sample rates of environmental microbiology are *Staphylococcus*, *Bacillus*, and *Corynebacterium* genus. The *Staphylococcus* and *Corynebacterium* genus source is the human body, and they are pathogens that induce infectious diseases. *Staphylococcus aureus* is a conditional pathogenic bacteria that NASA attaches great importance to, and a crew needs treatment before flight who is checked up positive on nasal resistance of *S. aureus* bacteria, and its purpose is to reduce infectious disease [14].

Therefore, the research and detection of microorganisms on orbit have become an important part of the medical monitoring and security of a manned spaceflight to understand the risk of air, water, and food contamination in the cockpit. This research and detection also contribute to understand the similarities and differences of microbial populations in the ground and extreme environments such as the international space station. Besides, this research can help identify microorganisms that pose a risk to the health of the crew and that have been studied to increase toxicity and thrive in spaceflight and microgravity and to study how microbes adapt to extreme conditions in space and could provide new perspectives for individuals and groups to adapt to space environments.

## 3. An Overview of Microbial Detection Methods in Space Station

### 3.1. From Culture to Nonculture

The cultural method is the standard method for the detection of microorganisms in the ground environment and the orbit environment. Test procedure and limit standard are normative and mature. However, there are some limitations in the on-orbit application. Firstly, the culture is the expansion of microorganisms, which has the risk of biosafety. Secondly, the on-orbit culture analysis needs 2-7 days, and the real-time performance is poor. Thirdly, it is impossible to identify pathogenic bacterium species and implement targeted prevention and control measures. In addition, the cultural method requires a solid culture medium, which requires load, storage conditions, effective time limit, postculture treatment, etc., increasing the flight cost. NASA and other space agencies have been looking for technologies to detect microbes on orbit without culturing, and NASA's roadmap calls for such technologies to be available on orbit by 2020. The characteristics of cultural methods and noncultural methods are shown in Table 1.

#### 3.1.1. Cultural Methods

During the period on orbit, a researcher uses an air sampler, medium contact dish, rapid sampling device, and water collector to monitor the microorganisms, in the location of air, surface, and water, and to control the microbial level within the index range effectively. Microbe species and quantity are controlled mainly from the life safety point of view as an astronaut. According to the regulation of the international space station medical control requirement documents, on orbit, the bacteria and fungi quantity in the air should be controlled in 1000 cfu/m^3^ and 100 cfu/m^3^ and the amount of surface bacteria and fungi should be controlled in 10000 cfu/cm^2^ and 100 cfu/cm^2^. Microbial test results should not include *Mucositis branham* (*coccus*), *Dermatitis bacillus*, *Histoplasma capsulatum*, *JK type Corynebacterium*, *Neisseria*, *Salmonella*, *Streptococcus pyogenes*, *Coccidiomyces*, *Kojae*, and *Cryptococcus neoformans* [6].

*Air sampling and culture*: America uses the Microbial Air Sampler module [15] on the international space station, and Russia uses the Ecosphere kit Sampler [16]. By pumping air sampling device aerodynamic effect, air produces high-speed airflow through the slit including the suspended carrier ions in the air and meets with the medium surface at the same time, so under the action of inertial impact on the culture medium, microbial particles are collected, and then cultivated and counted, and colonies go downward for further research. Bacteria are inoculated in trypsin soybean AGAR medium; fungi are inoculated in char-dodo AGAR medium or other media. The bacteria culture dish is cultured at 37°C for 48 hours, and the fungi culture dish is cultured at 28°C for 5-7 days; then, they are transported back to the ground laboratory by a cargo spacecraft for identification.

*Surface sampling and culture*: the main sampling methods are the medium contact method and sampling stick smear method. The Surface Sampler Kit [17] provided by the American cabin adopts the medium contact method, in which the medium conducive to the growth of microorganisms is made into a rectangular nutritional pad, which is then loaded onto a PVC plate. The collection area of the contact piece is 25cm^2^. The components of bacterial medium tablets are pancreatic protein soybean AGAR medium, while the components of fungal medium tablets are Chablis glucose AGAR medium or rose AGAR medium containing chloramphenicol. Bacteria and fungi media are pressed on the collecting surface, and then, the media are encapsulated and cultured. The culture environment of bacteria is 37°C 48 hours, and it is 28°C 5~7 days for the fungi. This detection method can realize on-orbit culture and evaluation. The Surface Pipette Kit [15] used in the Russian cabin adopts the method of sampling stick smear. A surface swab is used to smear the surface of a 10 cm × 10 cm area on the internal structure and equipment of the capsule. After sampling, the swab is put into a plastic tube with buffer solution and brought back to the ground laboratory for cultivation and testing.

*Water sampling and culture*: EHS water testing kits were used on the international space station [15]. Through the adapter connection, different tanks of water will be collected, and then water is injected into microbial capture to cultivate and count, or the rapid *Escherichia coli* bag is used to judge whether there is water pathogenic *E. coli* by color reaction.

#### 3.1.2. Nonculture Methods

The cultural method is the main method for the detection and research of microorganisms on orbit. The research and improvement of the noncultural method for the detection of microorganisms on orbit have never stopped. At present, some noncultural methods have appeared in detection and research of microorganisms in the space station.

The fluorescence analyzer is a semiautomatic, real-time analysis technique, using a “bioluminescence” reaction to measure ATP levels in samples to evaluate microbial biomass. In the project Euro Mir95 on the Mir space station, one of the main purposes of the microbe monitoring experiment on the miriam-t2 space station conducted by the Italian space agency was to verify this simple and rapid on-orbit microbe detection technique. China's ShenZhou spacecraft also used an ATP detector to detect microorganisms on orbit.

**Figure 1:**
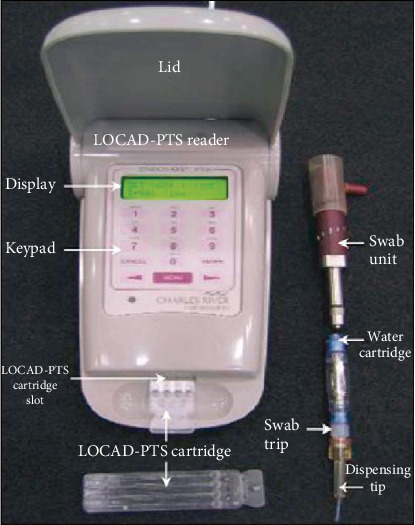
LOCAD-PTS microbial rapid detection installation.

“LOCAD-PTS” [6], a handheld microbial detection device, obtains microbial information by detecting characteristic biomolecules. The detecting system consists of a reader, a reaction box, a swabbing unit and swabbing kits, etc. (Figure 1) [17]. The sample solution reacts with a limulus reagent to produce color changes, and the number of bacteria is determined by the color changes after the reaction.

A simple portable system of gold nanoparticles could be used to detect microorganisms, and gold nanoparticles have a variety of properties, such as high stability, low toxicity, and photonic properties, which support their use in biological detection applications for human habitation in space [7]. The reflected color of the nanoparticles varies due to particle size and/or particle aggregation. Gold nanoparticles could be functionalized by covalent connection of various biomolecules, and conjugated functionalized gold nanoparticles by ligand conjugation could be used as chromogenic biosensors, in which the binding of the target and ligand leads to the aggregation and reddish-purple transformation of nanoparticles. NASA researchers are developing a bioassay to detect *Staphylococcus aureus*, and gold nanoparticles have been modified by covalent modification of antibodies that have a high binding affinity for proteins in the cell wall of *Staphylococcus aureus*. Preliminary test data from the gold nanoparticle bioassay system showed that *Staphylococcus aureus* could be detected within 10 minutes.

The microfluidic PCR instrument [8] is very suitable for space transportation of space life science research devices due to its features of miniaturization, low power consumption, small sample consumption, and high integration automation. In 2017, Chinese scientists used a microfluidic PCR instrument to study the mutation patterns of 20 genes in the space environment aboard the module of the international space station (ISS). At the 2011 NASA Johnson Space Center symposium, which presented a list of recommendations for long-term microbial surveillance missions, the group's consensus was that NASA should study and implement a molecule-based form of microbial detection, such as real-time PCR, that could work well in flight.

### 3.2. From Sequencing to High-Throughput Sequencing

Previously, due to the limitations of on-orbit sequencing conditions, molecular identification of microorganisms mainly relied on first-generation sequencing technology, including single-gene amplification sequencing of pure culture and PCR identification methods for identifying marker genes. In order to understand the status of cockpit microorganisms, some samples collected on orbit were brought back to the ground for depth detection using the second- or third-generation sequencing technology (Table 2). At present, some progress has been achieved in the research and application of on-orbit equipment. The microfluidic PCR instrument [18, 19] and the handheld sequencing instrument based on the third-generation sequencing technology [20–22] have been successfully operated on the international space station, marking the phased achievement of space sequencing. With the development of sequencing technology, high-throughput sequencing technology has been more and more applied in aerospace.

#### 3.2.1. First-Generation Sequencing Technology

In 1977, Sanger and Coulson proposed the end termination sequencing method of dideoxynucleotide (Sanger-Coulson method or Sanger method) and invented the first-generation sequencing technologies. The Sanger method, with a reading length of up to 1,000 bp and a high accuracy of 99.999%, has helped people complete a lot of sequencing work and remains the gold standard for sequencing. The first human genome map completed in 2001 was based on the improved Sanger method. In addition, the first-generation sequencing technologies include chemical degradation. Previous tests of on-orbit microbial samples mainly used first-generation sequencing technology to sequence the single gene amplification of pure cultures. Then, sequences were compared with the reference sequences in the database to determine the microbial species information, and the PCR identification methods were derived based on specific microbial gene sequences. Microfluidic technology makes on-orbit sequencing or PCR possible. Lab-on-a-chip is based on the technology of the microfluidic chip. Microfluidic is a kind of precise control and manipulation technology of the microscale fluid in the micro-/nanoscale. Microfluidic has the basic function such as biological, chemical laboratory, including sample preparation, reaction, separation, and detection of miniature in a few-square-centimeter chip, and its basic characteristics and advantages are unit techniques in the overall control of small flexible combination and scale integration platform. A microfluidic chip is very suitable for space life science research equipment due to its features of miniaturization, low power consumption, small sample consumption, and high integration automation. Microfluidic technology could be used to develop a small space sequencing instrument, PCR instrument, and sequencing preprocessing device.

In recent years, more and more microfluidic chip devices have been applied in space life science research. In 2006, the United States brought the lab-on-a-chip portable test system to the international space station via discovery. The lab-on-a-chip consists of a handheld diagnostic device and a small probe to help astronauts conduct biological research on issues ranging from astronaut health to the spacecraft's environment. In 2009, the international space station used microarray chips that detect gram-negative bacteria and fungi to detect microbes in the spacecraft, and microfluidic chips such as space cell culture and space protein detection had also been increasingly used in space. In 2011, the microfluidic chip gene amplification device of Beijing Institute of Technology was successfully carried on board the ShenZhou Eight and successfully completed the space biology experiment research, achieving the breakthrough of “zero” in the field of China's microfluidic chip space application technology. It was the first time for China to carry out a gene experiment in the space environment and realize on-orbit detection. In 2017, Chinese scientists mounted a microfluidic PCR instrument on the module of the international space station to study the mutation regularity of 20 genes in the space environment [21].

#### 3.2.2. Next-Generation Sequencing Technology

Next-generation sequencing technology, characterized by the sequencing of millions upon millions of DNA molecules at once, is a revolutionary change from conventional sequencing, also known as high-throughput sequencing. The next-generation sequencing technology has greatly reduced the cost of sequencing while greatly increasing the speed and maintained high accuracy of sequencing. According to the application of high-throughput sequencing technology, it can be divided into de novo sequencing, resequencing, whole transcriptome resequencing, metagenomic sequencing, targeted amplicon sequencing, and so on. Metagenomic sequencing and targeted amplicon sequencing technologies are increasingly used in microbiology, providing a more comprehensive molecular approach to the study of microbial diversity. Although sequencing technology has been shown to work on the space station, it has not been widely used for microbial monitoring on the international space station due to the special environment of the space station and the limitations of the station's payload capacity. Once simple, compact, and reliable sequencers and sample processors suitable for microgravity environment are developed, they will be able to be used for rapid and real-time microbial detection and functional analysis in the space station, with great potential in long-term on-orbit flight [23].

Through amplified gene sequencing of the 16s rRNA gene or eukaryotic internal transcriptional interval (ITS) region, comparing with the database, the microbial composition and classification status information can be obtained accurately, which has become a relatively mature method for the study of the microbiome. To monitor flora of the space environment and human, high-throughput sequencing technology can carry out in-depth investigation, which is conducive to monitor dynamic changes of flora and risk assessment. Many sequencing companies have begun developing new, portable, and miniaturized sequencers and sample processing devices, despite the fact that sequencers and associated sample processing equipment are too large to operate on orbit currently.

Many companies that make large sequencers are also moving toward miniaturization. In September 2015, ThermoFisher expanded its line of NGS sequencers for the first time following the acquisition of LifeTech, launching the new IonS5 series sequencers. Illumina released the first mini sequencer, the MiniSeq, in early 2016, and another mini sequencer, the iSeq™ 100 (covering nearly 1 square foot), in early 2018. BGI introduced BGISEQ-50, and BGI introduced MGISEQ-2000 and MGISEQ-200, respectively, in 2017. While these second-generation sequencer products are still a long way from portable specs, it is amazing to think about the history of computing, from the 26.5 m^3^ ABI prism 310 to the size of today's microwaves.

### 3.3. Third-Generation Sequencing Technology

Single-molecule real-time sequencing (SMRT) of Pacific Biosciences company and single-molecule nanopore technology of Oxford Nanopore company represent the third-generation sequencing technology, which is new sequencing technology containing advantages of high throughput, quick speed, long reads, and low cost. The characteristic is single-molecule sequencing and reduces the size of our equipment. The disadvantage of single-molecule sequencing is the high error rate, usually at around 15%. By increasing the depth of sequencing and using correction software, the accuracy rate can be 99.9%. MinION, a handheld sequencer based on third-generation sequencing technology, was taken to the international space station [22], where NASA astronaut Kate Rubins sequenced DNA samples of mice, viruses, and bacteria prepared on the ground in 2016. This means that astronauts will be able to test for genetic material or genetic variations directly in flight, by which NASA describes as ushering in a new era of gene sequencing of living creatures in space.

**Figure 2:**
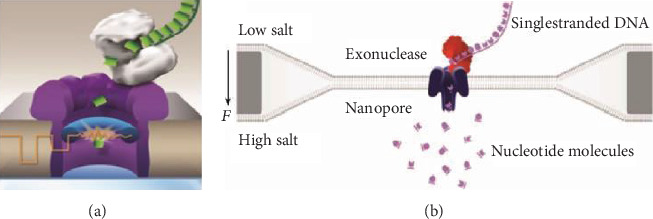
Nanopore DNA sequencing using electronic signals as detection methods. The diameter of the nanoscale is very small, and only a single DNA molecule is allowed to pass through. When a single strand of DNA passes through, it blocks the flow of ions and changes the current intensity across the nanopore. Because the charge properties of the four bases of ATCG are different, the type of base passed is identified according to the change property [25–28].

The MinION sequencer, called pocket sequencer, is just four inches long and consists of a sensor chip, specialized integrated circuits, and a flow control system for a complete single-molecule induction test. Unlike the current mainstream sequencers, MinION is a third-generation sequencer that uses a technique called nanopore sequencing. A special lipid bilayer containing a pair of electrodes on one side is placed over a micropore that contains a number of nanopores made up of hemolysin proteins, each of which binds to an exonuclease. When the DNA template enters the pore, the nucleic acid exonuclease in the pore will “grasp” the DNA molecule and cut off the DNA bases that pass through the nanometer pore in order. When each base passes through the nanometer pore, a block will be generated. According to the change of blocking current, the corresponding base types can be detected, and the DNA molecule sequence is finally obtained [24] (Figure 2).

## 4. High-Throughput Sequencing Technology and Applications

High-throughput sequencing, also known as next-generation sequencing (NGS), is marked by the ability to sequence millions upon millions of DNA molecules in parallel and by the fact that the average read length is short [29, 30]. NGS is relative to the first generation of sequencing technology–Sanger sequencing. The first generation of sequencing technology with longer reads and high accuracy is suitable for new species gene late leader from the construction of the framework and fills the GAP. But there are disadvantages of expensive, low flux, slow, and difficult to sequencing trace DNA [31]. High-throughput sequencing technology is the most widely used sequencing technology in current genomics research, which effectively avoids the tedious cloning process of the first-generation sequencing technology and has the advantages of high throughput, high speed, and low cost [32]. It has been increasingly used in the research of microbiome, which makes the research of microbiome produce a qualitative leap. It make us analyze the microbial structure composition, gene function, and metabolic pathway of the microbial ecosystem accurately and deeply. In the microbiological testing and research, frequently using the target gene amplification sequencing technology, through particular gene (e.g., 16s rRNA or ITS) amplification and sequencing, to get the sample data of bacteria and fungi in microbial diversity, the detection method generally includes four steps: DNA extraction, library construction, sequencing, and bioinformatics analysis.

Currently, the mainstream high-throughput sequencing technologies include Roche's 454 pyrosequencing technology, Illumina's Solexa technology, ABI's SOLiD technology, and Life Technologies' Ion Torrent technology [33, 34]. Although these sequencing technologies have their own characteristics, they have many similarities in principle: (1) the target DNA is cut into small fragments; (2) a single small fragment of DNA molecule is bound to the solid phase surface; (3) there is single-molecule-independent amplification; (4) only one base (A,C,T,G) is copied at one time, and the signal is detected; and (5) it is a high-resolution imaging system. High-throughput sequencing, with its high output and high resolution, not only provides us with rich genetic information but also greatly reduces the cost and time of sequencing. Among them, 454 pyrosequencing and Solexa are commonly used in microbiome research. The 454 pyrosequencing technology has the advantages of high precision, high throughput, high read length, and low cost [35]. The advantages of Solexa technology are the short sequencing cycle, convenient sequencing process, and more conduciveness to in-depth exploration of gene annotation and gene function. Second-generation sequencing has lower cost and higher accuracy, so the research of microbiome mainly uses second-generation sequencing technology.

### 4.1. High-Throughput Sequencing Ground Applications

Since the beginning of the human genome project, high-throughput sequencing technology has played an important role in analyzing the structure and function of human genes. Subsequent transcriptome studies have provided us with new ideas for exploring the pathogenesis and intervention measures of diseases such as tumors. High-throughput sequencing technology is widely used in food, medicine, environment, human, and animal microecology as well as other fields. In the microbiology field, microbial research is widely used in human pathogenic microorganism detection, the traditional fermented product study such as wine, vinegar, and traditional dairy products, environmental microbiology, and human body microecological research. The study of human microecology is a hot topic in recent years, which discovers “brain-gut axis” and other new findings [36–38], including correlation between intestinal flora and Alzheimer's disease, obesity, diabetes, and other diseases, and reveals the relationship between the body's metabolism and nervous system from the aspects of microbial groups. According to the research of human microecology, the human body is the host of a complex and rich microbial community, which is mainly distributed in the skin, oral mucosa, reproductive tract, and gastrointestinal tract and is the basis of health and disease. Normally, the flora of the human body is in a balance state, which has many benefits to the human body; for example, it can occupy the ecological niche to resist pathogen invasion, stimulate human immune system, produce beneficial biological active substances, and so on. When this balance is broken by certain factors, disease bacterium and conditional pathogen can take the opportunity to invade and cause harm to the airframe. Monitoring changes in the quantity or structure of microorganisms in the environment and human body through high-throughput technology can reveal the impact of the environment on human flora and human health. By analyzing microbial components of home, office, classroom, museum, and hospital environment through high-throughput sequencing technologies, scientists have revealed the microbial structure including bacteria, fungi, viruses, and protozoa, and certain microorganisms influence our health through our susceptibility to infectious diseases and allergy. High-throughput sequencing technology has led many scholars to innovate from quantity to quality in the research of various living objects and has broadened the scope and depth of genetic information research.

### 4.2. Application of High-Throughput Sequencing in Aerospace

#### 4.2.1. Detection of On-Orbit Environment Samples by High-Throughput Sequencing Technology

Although the station has been shown to be capable of experiments such as microbial culture, PCR, and sequencing, due to lack of practical instruments, the current station is still based on the simple cultural method of counting, and the station's microbial samples are mainly descended to the ground for microbial identification, high-throughput sequencing, and analysis.

*(1) Mir Space Station*. High-throughput sequencing is a research method that has been developed and widely used in the past ten years, and the cultural method was mainly used in the early microbial investigation and research on the Mir space station. The survey of Russian early Salyut and the Mir space station found that a manned spacecraft environment microbial ecosystem is the main characteristic of pathogenic bacteria of periodic accumulation, typical conditional pathogenic bacteria in the system, and stability in each niche showed dilated, until serious pollution is made and cleared off [12].

Three samples of condensed water from the back of the Mir panel were collected and returned to the ground for testing. Using a variety of culture techniques, a variety of organisms were cultured, including *Escherichia coli*, *Serratia marcescens*, and a presumed *Legionella* species. In addition, microscopic analysis indicated the presence of protozoa, dust mites, and spirochetes [39].

*(2) International Space Station*. The international space station, currently running, a narrow human habitat, has been inhabited since November 2000 and contains a large amount of microbes concomitant with the human on the international space station. Test samples from air and surface contain abundant *Staphylococcus*, *Bacillus*, and *Corynebacterium*. *Penicillium*, *Aspergillus*, and *Cladosporium* are the most common fungi. The most common microorganisms in the water samples were *Ralstonia pickettii* and *Burkholderia multivorans*. *Staphylococcus* and *Corynebacterium* are mostly bacteria from the human body. Although many microorganisms have been isolated and cultivated, the number of bacterial strains obtained by the cultural method is limited, and a large number of unculturable microorganisms cannot be analyzed statistically.

As part of the NASA's plan, based on qPCR, 16s rRNA gene amplification sequencing, and ITS regional amplification sequencing, microbial composition on the surface of the international space station is tested, and Enterobacteriaceae is more than 50% in total testing sequences of 24 samples, followed by *Bacillus* (about 13%) and *Staphylococcus* (about 10%) [23]. Interestingly, the international space station environmental microbes, especially the most abundant groups, will not change with the change of time and place. During the flight of three missions on the ISS, all the relative abundance of the microbial taxa is different, but difference between eight places of each mission is not significant. At the genus level, a total of 121 taxa are identified, of which 77 can be attributed to known genera, 68% are known human microbial constituents, and the remaining 32% are found in environments such as soil and water. The microbial communities of the international space station mainly belong to Staphylococcaceae, Corynebacteriaceae, Caulobacteraceae, Pleosporaceae, and Sporidiobolaceae [39, 40]. *Pantoea*, *Methylobacterium*, *Penicillium*, *Rhodotorula*, and *Rhodotorula* have higher abundance [14]. Bacteria related to human skin, such as *Staphylococcus*, is widespread in living environments, in line with terrestrial simulations. Lang et al. [41] analyzed 15 samples on the ISS surface by 16s rRNA high-throughput sequencing technology and found that the surface microbial community composition on the ISS was close to the surface of the human activity environment. 16s RNA gene and ITS high-throughput sequencing technologies are widely used in different samples of ISS, provide information about bacterial and fungal community composition, and show that an astronaut is the main source of microbiota in the international space station [42]. In most cases, the recognition of bacteria is thought to be associated with the human, especially the skin.

Samples were collected from three locations (air diffuser, handrail, and surface) of the Japanese experimental module KIBO on the ISS, and the bacterial and fungal biota were analyzed using quantitative PCR and high-throughput sequencing technology. Proteobacteria and Firmicutes are detected on the inner surface of KIBO, in which Staphylococcus and Enterobacteriaceae bacteria dominate, and most of the detected bacteria belong to the human microbiome [43]. Using prelaunch samples as controls, *Chigospora* and *Malassezia* were the dominant bacteria in prelaunch and space, respectively. The dominant species found in the air conditioner diffuser, lab bench, door push panel, and facility surface specimens were *Inonotus* sp., *Cladosporium sp*., *Malassezia sp*., and *Pezicula* sp. Fungi in KIBO may come from human contamination [44].

Water recovery and conservation are key issues for the survival of the space environment. In addition to the recovery system, water quality index testing methods including microbial load are also needed to evaluate the microbial load and storage methods of long-term drinking water. Bacci et al. [45] manage drinking water of Russian cosmonauts on ISS, with fluoride, colloidal silver, and silver ions in several different methods. Stored five years, the bacterial abundance sequencing analysis using 16s rRNA amplification showed the differences between different disinfectants and proved that different disinfectants for water microbes have selective effect. *Proteus* is ubiquitous in all samples, which is consistent with the research reports on microbial community composition in the water bodies of the earth [46].

In conclusion, ISS environmental flora is affected by human activities, and dominant bacteria such as *Staphylococcus* exist in large quantities in human skin. Under isolation conditions, the flora structure is still a highly dynamic system, which can adapt to the habitat of the closed chamber. Since the space station cannot achieve a sterile environment, it is necessary to conduct dynamic bacterial community monitoring to avoid the increase of high antibiotic resistance or potential pathogens and the decrease of bacterial diversity. In the future, it may be necessary to select appropriate microbial monitoring methods and possible countermeasures to ensure the safety of microorganisms in the spaceflight environment.

#### 4.2.2. Human Microbial Research of On-Orbit Samples by High-Throughput Sequencing Technology

In April 2019, the result of the NASA twins experiment project was published in the journal *Nature*, in which the intestinal microbial genome and metabolome of on-orbit and ground-based control twins were tested and found that the flora abundance of on-orbit pilot (TW) was lower than that of ground-based control (HR) [47]. Skin fungi flora of the 10 astronauts who stayed on the international space station was analyzed with pyrophosphate sequencing and quantitative PCR technology, and the result was that Marla bacteria diversity decreased after the flight. In the 10 astronauts, ascomycetes were detected in five samples in the flight, and accident adhesion on the skin may occur before the flight and continue to adhere to the skin after flight [48]. This observation indicates the ability of a particular or uncommon microorganism to proliferate in a closed environment. Immune systems of human exposed to stress and extreme environmental conditions during spaceflight were weakened, resulting in increased susceptibility [49, 50]. At the same time, through the adaptation process to extreme conditions, the bacteria showed stronger toxicity [51, 52] and lower sensitivity to various antimicrobial agents [53, 54]. It has been reported that *Staphylococcus* and *Enterococcus* isolated from the international space station have more resistance genes than those from the ground [55]. In fact, there have been up to 29 cases of urinary tract infection and subcutaneous skin infection during spaceflight, with the more serious cases being superficial skin infection [1]. Skin microbe is not only the main source of microbe in the aircraft cockpit but also an important factor affecting skin health. As the first line of defense of the body's immune system, the natural bacteria of the skin occupy the ecological niche and can help us resist foreign pathogenic bacteria. However, when the natural bacteria of the skin are out of balance, some opportunistic pathogens may make the skin more vulnerable and cause serious infections.

#### 4.2.3. Application of High-Throughput Sequencing Technology in Ground Simulated Space Environment

The space environment is a relatively closed system with no exchange with external air, and these environmental factors have an important impact on microbial dynamics and flora characteristics. In order to monitor the microbial characteristics of the closed system on the ground in real time, researchers designed different experiments and simulated buildings to conduct a series of studies on the microorganisms in the air, surface, water, and human body in the closed environment.

In confined space, air cannot be exchanged with the outside world. Microbial pollution in the air is an important monitoring index and research object. An isolated scientific research station of the Antarctica Halley station had been researched of microbial diversity in air, to identify potential sources of microbial populations. Using high-throughput sequencing, it was found that many DNA sequences were obtained from nonculturable microorganisms, and the biodiversity in the air between the summer and winter had no obvious regularity [56]. Molecular technologies, along with traditional microbiological methods, were utilized to catalog microbial succession during a 30-day human occupation of a simulated inflatable lunar/Mars habitat. Next-generation sequencing was used to elucidate the microbial dynamics and community profiles at different locations of the habitat during varying time points, and the results of this study revealed a strong relationship between human presence and succession of microbial diversity in a closed habitat [57]. The environmental airborne bacterial population in relation to human confinement was investigated over a period of 1 year in the Concordia Research Station, which was located on the Eastern Antarctic plateau. The researchers found that the total bacterial contamination increased over time during confinement but diminished after reopening of the base [58]. The Mars500 project was conceived as the first full duration simulation of a crewed return flight to Mars. For 520 days, six crew members lived confined in a specifically designed spacecraft mock-up. Microbial composition analysis showed that *Staphylococcus* dominated in the air, and areas with high human activity were identified as hotspots for microbial accumulation. Previous studies showed that *Staphylococcus* was widely distributed in human skin and respiratory tract and was a biomarker for human activities in various indoor environments [59–61]. The number of *Staphylococcus* and *Corynebacterium* increased during the isolation period [57] and decreased after the isolation, which was consistent with the previous survey results [56–58]. The composition of environmental microorganisms is related to the location of the environment and the living time of the volunteers. Human beings are important vectors of microorganisms in the built environment and have greater influence in the more closed environment [62–64].

The enclosed environment is conducive to the spread of microorganisms, affecting human health, while surface microorganisms are important factors in the corrosion of spacecraft materials. Microbial component analysis of the surface sample of Mars500 found that main dominant fungi belong to Firmicutes, Proteobacteria, Actinobacteria, and Bacteroidetes, and in the genus level, the dominant bacteria are *Staphylococcus*, *Corynebacterium*, *Enterobacter*, *Micrococcus*, *Pseudomonas*, *Bacillus*, and *Staphylococcus* [57].

Silver ions are the commonly used disinfectants of drinking water in space. Using Illumina MiSeq high-throughput sequencing technologies, researchers analyzed bottled water dealt with different concentrations of active silver ion for 60 days, to observe the effects of silver ions on disinfectant microbial species diversity. The results showed that the dominant bacteria are Proteobacteria, Firmicutes, and Actinobacteria. The study has confirmed the antibacterial effect of active silver ions, providing a basis for ensuring the safety of astronauts' drinking water during spaceflight [65].

Space microbial studies began in the Apollo program in the 1960s. The crew body microbes were tested before and after flight, and microbe migration between the crew was found. Subsequent research had shown that exchange of pathogenic microorganisms between crews occurred mainly in more than 18 days of mission. The harm of bacterial migration is undoubtedly to break the balance state of the passengers. Therefore, the study of the status of the passengers' microbial population is conducive to the prediction and prevention of the occurrence of infectious diseases on orbit. China has carried out the 180-day controlled environmental and life support system (CELSS) experiment [66]. Microbiological samples from four crew members (including the forehead, ears, chelidon, armpits, and groin) were collected before the experiment and 1 month, 2 months, 3 months, 4 months, and 5 months in the tank and finish time, respectively. Using 16s rDNA V3-V4 high-throughput sequencing technologies for flora structure analysis, the result showed that surface species mainly belonged to Actinobacteria, Firmicutes, Proteobacteria, and Bacteroidetes. In the genus level, *Corynebacterium* and *Staphylococcus* were dominant in five parts. Dynamic analysis showed that the bacteria after the first month of community changed significantly compared with the month before the cabin entry and then gradually returned to a steady state, suggesting that closed environment as a stress factor can affect the body surface bacterial community structure [67–69]. Russia conducted a simulated human 520-day (Mars500) capsule landing experiment on Mars, using high-throughput sequencing technology to study the dynamic changes of the intestinal flora structure over time in six volunteers [70]. The study found that in a tightly controlled closed environment, the human intestinal flora was dynamic, and the changes of intestinal microflora were highly individual, but some key microbial components showed stability, which had potential significance for maintaining the mutually beneficial microbial structure of the intestinal tract.

## 5. Prospects, Problems, and Possible Improvement on Sequencing Applications in Space Microbiology

### 5.1. Application Prospects of High-Throughput Sequencing Technology on Space Microbiology

With the expansion of the scope of human exploration, deep space exploration may take years or decades on future flight, and that may put higher requirements forward for the space life science research system. Space life science research is limited by the payload carrying capacity of space vehicles, so conventional laboratory instruments and equipment cannot meet the requirements of space carrying due to its large size, high power, large sample consumption, complicated operation, and low functional integration. The instrument suitable for on-orbit detection needs to meet the characteristics of miniaturization, low power consumption, small sample consumption, and high integration automation. Therefore, the integration and intelligentization of instruments and technologies in the space field are the future development trend.

In the future research and application of space microbiology, the traditional and complicated methods of microbial culture and identification have been unable to meet the needs, so it is necessary to establish a microbial identification method that can operate independently, easily, and rapidly. The handheld sequencer based on the third-generation sequencing technology [22] has been successfully operated in the international space station, which marks the phased achievements and great application potential of space sequencing.

Known as a pocket sequencer, MinION is only 4 inches long and about the size of an ordinary usb flash drive. It uses nanopore sequencing technology and consists of a sensor chip, an ASIC, and a complete flow control system for testing single molecules. MinION was taken by NASA to the international space station, where DNA samples of mice, Escherichia coli, and lambda phages ground-prepared were sequenced, by which NASA described as the beginning of a new era of human genome sequencing in living organisms in space. Mice, Escherichia coli, and lambda phage, respectively, represent the eukaryotes, prokaryotes, and virus. The experiment proved feasibility of sequencing analysis and microbial identification on the international space station and illustrated application potential of the high-throughput sequencing technologies in the space, including the microbiological assay, disease diagnosis, environmental monitoring, and the molecular basis research of biology in space.

From the perspective of spaceflight, in the short run, on-orbit applications of high-throughput sequencing technology will greatly speed up research in the space station by allowing researchers to obtain real-time on-orbit data without waiting for samples to return; microbial identification in flight can be carried out to understand the changes of the microflora structure and pathogenic microorganisms on the space station, so as to prevent the occurrence of infectious diseases. For the personnel with infectious diseases, on-orbit analysis can provide a basis for treatment, including the use of targeted antibacterial drugs. On-orbit metagenomic sequencing enables researchers to understand the mutated strains in the space station in real time and prevent the growth and reproduction of harmful mutated microorganisms. To illuminate molecular mechanisms of microbial adaptation in the space environment, on-orbit sequencing technology makes gene expression research possible in-flight, and avoids the storage and degradation of unstable RNA, so we can perform experiments more stably, and reduces the risk of experiment failure. Interestingly, on-orbit sequencing makes it possible to explore life in deep space and discover extraterrestrial microbes.

### 5.2. Possible Models and Methods of Future Sequencing Applications in Space Microbiology

According to the standard sequencing operation procedure on the ground, sample preparation before sequencing includes DNA extraction, Polymerase Chain Reaction (PCR), library construction, and other steps. After sequencing, special analysis software and database are needed for interpretation. These cannot be done as easily on the space station as it is on the ground, so some process optimization needs to be done before high-throughput sequencing can be applied on orbit independently.

Sample preparation requires an automated sample preparation system that converts biological samples into a form that can be sequenced, requiring small size, simple operation, stability, and reliability, so that efficient and simple DNA extraction methods are needed. Microbial DNA can be extracted by simple thermal cracking and magnetic bead adsorption, which is simple and independent of gravity. For the sample preparation of the MinION sequencer, Oxford developed a simplified sample preparation method, for example, Oxford Nanopore Technologies 1D rapid library preparation kits and VolTRAXTM automated sample preparation device, and they are being optimized for different samples.

PCR, which duplicates DNA in front of sequencing, takes longer time, and the designed primers are easy to cause species bias. Including the nanopore sequencing technology and single-molecule real-time sequencing technology (SMRT), the third-generation sequencing technology can be used to sequence DNA sample directly, without PCR, and it is the most possible on-orbit sequencing technology model in the future.

For data processing, intervention from earth during a deep space mission will be limited to electronic communications only, as the processing of the sequenced raw data will require a portable, highly computational computer to analyze and process the data. With more deep space communication delays and less data transmission rates, local analysis of sequencing data will become critical.

### 5.3. Existing Problems and Expected Improvement

High-throughput sequencing technology shows the potential of microbial research in space. However, there are some problems needed to be improved. The accuracy of the third-generation sequencing technology needs to achieve higher sequencing accuracy by reducing the number of false-positives and false-negatives to improve the diagnostic capacity. We can improve the sequencing accuracy by improving the resolution of nanopores and optimization algorithm [71]. For example, after nearly 10 upgrades to MinION, the minimum error rate for the latest version has dropped to 2%, compared with 0.1% for Illumina sequencers. Assuming sequencing accuracy comparable to that of the Illumina sequencers, the third-generation sequencers could play an important role in the study of microbes in space.

## 6. Conclusions

On-orbit sequencing technology has great potential for development. Through real-time on-orbit detection of microorganisms, the changes of the microbial community structure and pathogenic microorganisms in the space station can be immediately understood, which is conducive to the prevention of infectious diseases. The on-orbit analysis can provide the infection status of bacteria or virus for the on-orbit personnel with infectious diseases and provide the basis for timely treatment. Through on-orbit metagenomic sequencing, we can also understand the situation of mutated strains in the space station in real time and prevent the growth and reproduction of adverse mutated microorganisms in time; on-orbit sequencing is of great significance to human exploration of deep space life and discovery of extraterrestrial microorganisms.

To realize on-orbit sequencing, on-orbit detection equipment and on-orbit sequencing analysis technology are the bottleneck problems to be solved first. Special environmental factors in space, such as vacuum, high temperature, low temperature, weightlessness, vibration, and cosmic radiation, not only have special requirements on experimental methods and operations but also have more stringent requirements on the design, weight, automation and integration, impact resistance, and other aspects of equipment for space life science research. Space life science research is limited by the payload carrying capacity of space vehicles. Conventional laboratory instruments and equipment cannot meet the requirements of space carrying due to large size, high power, large sample consumption, complicated operation, and low functional integration. According to the standard operating procedures on the ground, the sample preparation before targeted amplicon sequencing includes DNA extraction, PCR amplification, and other steps, and raw data need to be interpreted by special analysis software after sequencing, and these steps cannot be completed independently in the space station currently. On the space station, an automated sample preparation system is needed to convert biological samples into a sequenceable form, requiring small size, simple operation, and stability. The high error rate is a problem encountered by the third-generation sequencing technology. In addition, the analysis of sequencing data requires a small and powerful computer, which requires its own database and analysis software. Only by solving these problems can on-orbit sequencing be achieved. Companies like Oxford are already doing that. With the rapid development of microfluidic technology, third-generation sequencing technology, and artificial intelligence, as well as the urgent demand for extraterrestrial space exploration and on-orbit sequencing technology by various aerospace powers, I believe these problems will be solved soon. Because there are no on-orbit sequencers for technical reasons, many on-orbit samples can only be brought to the ground for genetic sequencing. With the rapid development of science and technology, the continuous research and improvement of microsequencing instrument and sample processing device, it is believed that there will soon be a suitable sequencing device for use on orbit, adding a sharp tool for the human space industry and the exploration of extraterrestrial civilization.

## Figures and Tables

**Table 1:** Comparison of cultural methods and noncultural methods.

	Object	Methods	Advantages	Limitations
Cultural methods	Air Surface Water	The air sampler package Medium contact method Water testing kits	Ability to monitor microbial populations on orbit	Long analysis period, the existence of unculturable microorganisms, risk of recontamination
Noncultural methods	Air, surface, and water	The fluorescence analyzer; LOCAD-PTS	Fast	Unable to identify microbial species
		Microfluidic PCR	High accuracy, fast	Able to identify known species, unable to identify unknown species
		High-throughput sequencing	Fast, high throughput, and high accuracy	Lack of sequencers on orbit, generally analyzed on the ground

**Table 2:** Comparison of three generation sequencing technologies.

	Principle of sequencing	Technical platform	Read length	Advantages	Limitations
The first generation	Chain termination method	Sanger	1000 bp	High accuracy, long read lengths	Low throughput, high cost, and low efficiency
The second generation	Pyrosequencing, sequencing by synthesis, sequencing by ligation	Roche/454, Illumina/Solexa, ABI/SOLiD	150-300 bp	High throughput, high accuracy, and low cost	Short read lengths, resulting in difficulties in repetitive/homopolymer regions, subsequence data analysis and genome assembly
The third generation	Sequencing by synthesis (DNA polymerase), electronic signal sequencing (exonuclease)	PacBio SMRT, nanopore	2-10 kb	Longest average read length, no amplification of sequencing fragments, portable	Low accuracy, dependence on DNA polymerase or exonuclease activity
